# Single-generation effects on terpenoid defenses in lodgepole pine populations following mountain pine beetle infestation

**DOI:** 10.1371/journal.pone.0196063

**Published:** 2018-05-14

**Authors:** Sharleen L. Balogh, Dezene P. W. Huber, B. Staffan Lindgren

**Affiliations:** Natural Resources and Environmental Studies Institute, University of Northern British Columbia, Prince George, British Columbia, Canada; Instituto de Biologia Molecular y Celular de Plantas, SPAIN

## Abstract

The recent mountain pine beetle outbreak in western Canada provides an opportunity to study the selection and heritability of tree defenses. We examined terpenoid-based defenses of seedling lodgepole pines which were offspring of mature trees subjected to high levels of mountain pine beetle selective pressure. Seedlings were grown from one of three types of cones: old cones on live trees; young cones on live trees; and cones on trees killed by beetles. Offspring thus represented crosses of non-surviving (NS) x surviving (S), S x S, and NS x NS parents, respectively. Methyl jasmonate was used to induce a defensive reaction in the seedlings. Seed source had a significant effect on levels of ten different terpenes, but not on total terpene concentrations. When the seedlings were grouped by location and treatment type, the seedlings of different cone types could be almost entirely distinguished by terpene profiles.

## Introduction

The mountain pine beetle (MPB), *Dendroctonus ponderosae* Hopkins (Coleoptera: Curculionidae), is the most destructive insect pest of lodgepole pine, *Pinus contorta* Dougl. var. *latifolia* Engelm. (Pinaceae), in western Canada, and has recently undergone the largest bark beetle outbreak in recorded history [1]. This outbreak has created substantial selective pressure on the lodgepole pine trees of the region. However, in spite of extremely high rates of mortality, including of immature trees [2], some mature trees survived the outbreak. This has created an ideal opportunity to study potential differences in defensive capabilities between those trees that survived the outbreak and those that did not, and whether these differences are heritable.

The MPB, like other species of bark beetles, colonize and reproduce within the inner bark of mature host trees. The first attack on a new potential host is initiated by a single pioneer female beetle, which selects a host, either by random landing [1,3, 4] or by primary attraction [4, 5], or they may use both depending on scale such as selection of a single tree vs. a forest patch [6]. After a pioneer female beetle enters the bark of a potential host tree, a signaling cascade is initiated to attract male and then further female beetles. This involves a number of both female and male produced pheromones, which, in conjunction with host volatiles, including myrcene and terpinolene, act to initiate a mass attack [7–10]. Concurrent with the mass attack, the beetles inoculate the host with associated blue stain fungi, primarily *Grosmannia (Ophiostoma) clavigera* (Robinson & Davidson) Zipfel, de Beer and Wingfield and *O*. *montium* (Rumbold) von Arx], which they often carry with them either in specialized mycangia or on their exoskeletons [1]. This strategy, when successful, ultimately overcomes the tree’s defenses and results in its death.

Lodgepole pines protect themselves against attacks, such as those by bark beetles, by the use of both constitutive and induced defenses [11]. These include chemical defenses, via increased production of secondary metabolites in the oleoresin [12–14]; and physical defenses via anatomical changes such as formation of traumatic resin ducts (containing defensive resin), cellular changes in the cambium layer [13, 15–17], and the formation and modification of stone cells [18]. Terpenes are the most abundant secondary metabolites within lodgepole pines, as they act as a physical and chemical barrier to seal wounds and are toxic and/or repellant to many pests. However, they can also serve as attractants for insects to locate or identify suitable hosts [11]. Defensive reactions within lodgepole pines and other conifers can be artificially induced by the application of the phytohormone, methyl jasmonate (in the form of MeJa, the volatile derivate of jasmonic acid), which causes an induced stress response in plants via the regulation of gene expression, to reallocate energy from photosynthesis and growth to secondary metabolite production [13, 16, 19]. Further, this MeJa-induced response produces many of the same anatomical changes in conifers as that of mechanical wounding meant to mimic bark beetle attack [16], although the defensive monoterpene response from MeJa is more generalized and quantitatively different than that of simulated bark beetle attacks consisting of both mechanical wounding and fungal inoculation [20].

Multiple enantiomers of some lodgepole pine monoterpenes may be present simultaneously within a tree, and may produce different effects on attraction or defense towards pests such as bark beetles [21–23]. Many bark beetle species, including both *Ips* spp., and *Dendroctonus* spp., show enantiomeric selectivity to the chirality of pheromone components, and the antipode to the appropriate enantiomer may have no behavioural effect, or a different effect [22, 24]. Further, the ability of bark beetles to produce enantiomers or isomers of pheromones appears to be influenced by the chirality of the precursor terpenes within their host trees. For example, the ability of several species of *Ips* spp. bark beetles to produce either (+)-*cis*- or (+)-*trans*-verbenol has been found to be dependent on the chirality of the volatile α-pinene that it is exposed to in its host tree. In the presence of (–)-α-pinene, the beetles produce (+)-*cis*-verbenol, which acts as the beetle’s biologically active pheromone, while in the presence of (+)-α-pinene, the beetles produce (+)-*trans*-verbenol [25, 26]. In addition, the western pine beetle, *Dendroctonus brevicomis* LeConte (Coleoptera: Curculionidae) appears to oxidize the enantiomers of α-pinene into the corresponding enantiomer of *trans*-verbenol, of which the (–) enantiomer is the biologically active compound, and is inhibitory to the positive chemotactic response of female beetles to other pheromone components [27].

The production of secondary metabolites within induced defensive resin, and thus resistance to the MPB and its associated blue stain fungi, varies dramatically between lodgepole pine individuals [28], and appears to be influenced by genetics [29]. Wallis *et al*. [30] found that when clones of lodgepole pine from different provenances were grown together, they had differing levels of secondary metabolite production, which affected resistance to several foliar pathogens. In addition, Ott *et al*. [31], found that there were heritable differences in overall as well as individual terpene production between half-sibling families of lodgepole pines. These genetic differences can lead to certain genetic resistance traits towards the MPB. For example, Raffa and Berryman [32, 33] found that lodgepole pine trees resistant to the MPB tended to produce higher quantities of induced monoterpenes when compared to susceptible trees, but quantities of constituent monoterpenes were similar. Further, Clark *et al*. [34] found that lodgepole pines from different geographic locations historically subjected to heavier MPB pressure may have lower levels of constituent terpenes than those with less historical beetle pressure. Finally, Cudmore *et al*. [35] found higher beetle reproduction in trees in areas with low historical climatic suitability for MPB. This may suggest that lower constituent terpene levels could make the trees less apparent to searching beetles and may thus effectively hide the trees from detection [34]. Therefore, it could be possible that the most resistant individuals would be those that had the lowest levels of constituent defenses, but the strongest induced defenses. Under the severe beetle pressure seen in British Columbia in recent years [2, 36], these trees may be the ones to preferentially survive, and have the opportunity to pass these traits on to their offspring. This study was therefore undertaken in order to determine:

1. If parental lodgepole pine trees with phenotypes that may have shown differential resistance to the MPB resulted in offspring that exhibited differential terpene secondary metabolite production.
2. If such an effect is detected, to characterize these differences based on the terpenoids affected.

## Materials and methods

The trees used for this experiment were seedling lodgepole pine trees of approximately three years of age, grown from seeds collected in the fall of 2011. Collected seeds were obtained from mature lodgepole pine in three geographic areas with varying levels of MPB-caused mortality. No specific permissions were required for these collections as the collection was of plants that were not endangered or protected.

**Fig 1:**
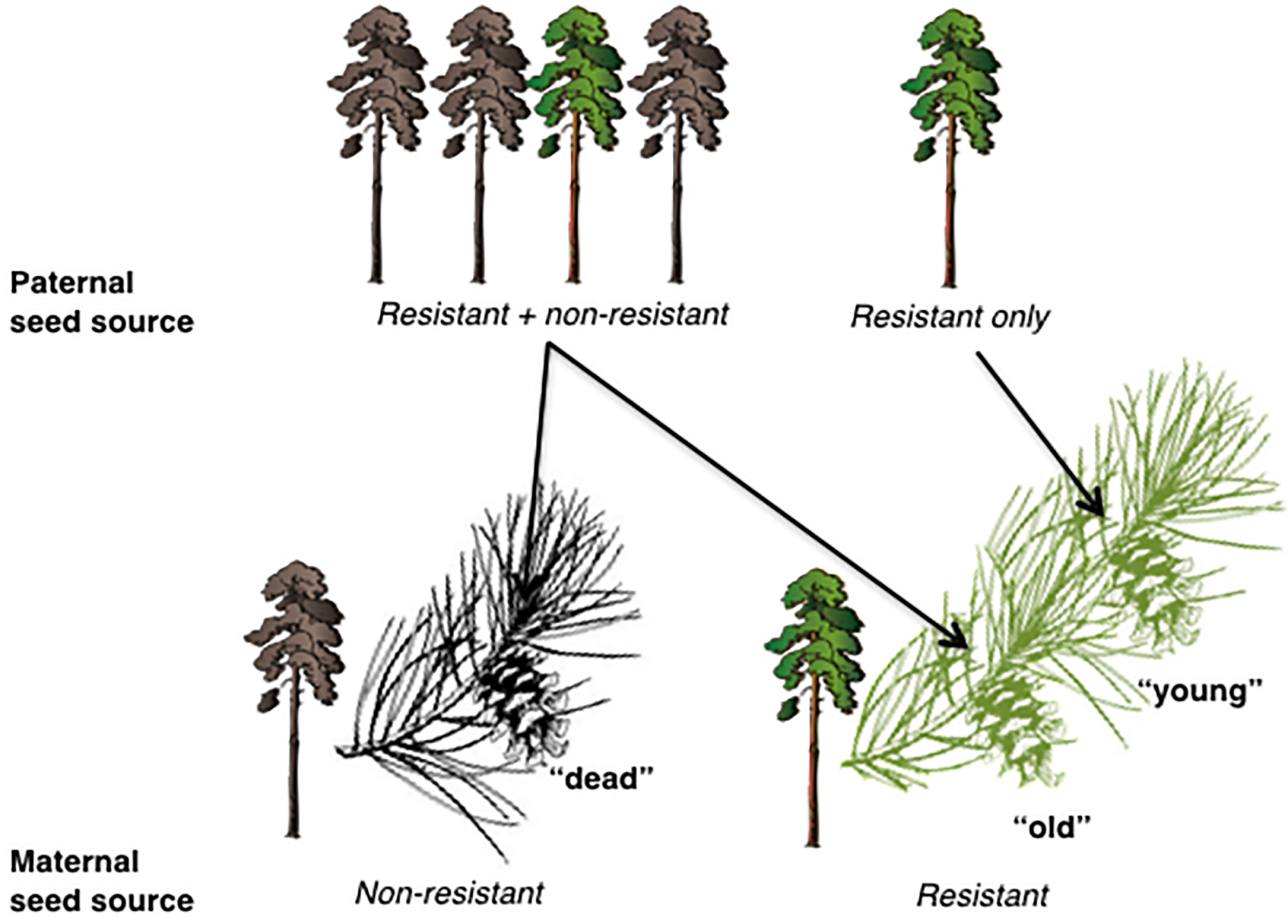
Illustration of paternal and maternal seed sources of cone types of lodgepole pine collected and grown in the I.K. Barber Enhanced Forestry Laboratory at the University of Northern British Columbia. The cones were collected such that there were three combinations of surviving (S) and non-surviving (NS) cones: “dead” cones = NS X NS, “old” cones = S X NS, and “young” cones = S X S.

At each site, cones were collected from a total of 10 live and 10 dead trees. Most lodgepole pines produce serotinous cones, which remain unopened on the tree until temperatures reach sufficient levels to trigger seed release [37]. Therefore, for the live trees, cones were collected from as close to the trunk as possible (“old cones”, for which cones were collected that would be old enough for pollination to have occurred before or early during the outbreak), as well as near branch tips (“young cones”). Thus, for the young cones, the maternal tree would have survived the MPB outbreak, and the cones would have likely been pollinated after the height of the outbreak and thus primarily pollinated by mature trees that also survived [surviving (S) x S]. However, for the old cones on the same trees, although the maternal tree would have survived the MPB outbreak, they would be more likely to have been pollinated prior to the main MPB outbreak by the entire population of trees, most of which did not survive the outbreak and therefore would presumably include trees that were more susceptible to the MPB. For the purposes of this study, this larger population of trees is described as “non-surviving” (NS) to indicate that a portion of the trees were of higher susceptibility, although this population of trees would be expected to also include individuals which survived (S X NS). Finally, cones collected from dead trees would likely have been pollinated by non-surviving and thus potentially less resistant trees, as well as taken from a non-surviving maternal tree (NS x NS). Therefore, the cones were collected in a manner to maximize potential differences in resistance traits of parental trees among the three types of cones collected. For an illustration depicting the seed sources and cone types, see Fig 1.

On 20 February 2012, 50 seeds collected from each cone type on each maternal tree were planted in Styrofoam G10223 SUPERBLOCK 112/80ML and grown in the I.K. Barber Enhanced Forestry Laboratory (EFL) at the University of Northern British Columbia. On 19 and 20 July 2013, seedlings were re-potted in standard one-gallon black round plastic pots (155mm X 175mm dimensions) in a mixture of peat: sand at a ratio of 2.7:1. Repotted seedlings were grown outdoors until used for experiments.

### Treatment of seedlings

A total of 150 seedlings were selected from the larger population of potted seedlings, grown as described above, for use in the experiment. “Families” of seedlings were designated so that each family consisted of seedlings grown from the same maternal tree. Seedlings were chosen such that a total of 10 families within the three sites were utilized (five from dead maternal trees, five from live trees). Families were chosen based on the germination success of their seedlings in order to achieve sufficient replication. Those families that had the most available healthy seedlings were selected, while keeping the distribution of families spread across the three sites as evenly as possible (Table 1). Within each of the families, one or two cone types were present (one cone type from dead trees and two cone types from live trees). Ten seedlings from each family and cone type were randomly selected, five each randomly designated as “treatment” or “control” seedlings. Any seedlings that were not in good health or whose stems were leaning more than 45 degrees compared to the soil surface were not used, and another seedling was randomly selected within the same family and cone type.

**Table 1:** Characteristics of sites and families used for experiments of induced terpene responses, planted and grown at the I.K. Barber Enhanced Forestry Laboratory at the University of Northern British Columbia.

Site	Family	UTM	Age	DBH (cm)	Height (m)	Tree Status	% Pine in Stand	% Live of Pine in Stand
McBride	3	5882849 0323803	29	24	7.8	Dead	50%	90%
	4	5882847 0323780	35	30	9.7	Alive	50%	90%
	7	5882086 0324725	27	26	9.2	Alive	80%	70%
Mackenzie	1	6120141 0497204	24	23	10.1	Alive	25%	95%
	3	6120305 0497609	24	25	8.5	Dead	30%	50%
	12	6126737 0486445	33	24	15	Alive	5%	100%
Carp Lake	2	6089157 0494399	106	30	21	Dead	41%	3%
	11	6072696 0492783	31	18	6.5	Dead	90%	90%
	15	6076281 0487849	30	19	4.6	Alive	40%	100%
	16	6076281 0488087	40	22	12	Dead	40%	70%

Percentages of pine in stand and live pine in stand are estimates, with “% live of pine in stand” representing live pine divided by live plus dead pine.

Two days prior to treatment with methyl jasmonate (MeJa), all selected seedlings were moved inside the Enhanced Forestry Laboratory (EFL) greenhouse at UNBC, watered thoroughly, and their heights measured. On 5 June 2014 –the day of the first application—control and treatment seedlings were moved into separate adjacent compartments in the EFL, with positions of the seedlings in 5x7 rows determined randomly, although the positions of the control and treatment seedlings were paired so that each position in its corresponding compartment had seedlings from the same site, cone type and family. Three seedlings in the control group were observed to have black aphids (most likely *Cinara* sp.) on their newly grown leaders. The aphids were removed by spraying them with high- pressure water, and the seedlings marked with flagging tape for further monitoring.

Seedlings were treated twice, on 5 and 19 June 2014 with either a MeJa solution (treatment group) or control solution (control group). Experimental seedlings were not watered for two days prior to and one day after application, to allow the solutions to absorb into the soil and prevent excessive runoff. Procedures for the treatment were adapted from Huber *et al*. [13]. Solutions of 1 L of deionized autoclaved water, with 1 mL of TWEEN 20 were prepared on the same day of application, in capped 1 L glass bottles. The treatment solutions had a further 100 μL of MeJa added. The MeJa solution was stirred until no visible droplets of the compound remained, about 1 hour. One hundred fifty milliliters of the appropriate solution was poured slowly as a drench into the pots of each of the treatment or control seedlings immediately next to the stem, as prior studies have shown that jasmonic acid-induced responses can be transferred between below- and above-ground plant tissues [13, 38]. The seedlings were then evenly spaced throughout the middle of the compartment, so that there was minimal contact between each seedling and its neighbours.

The temperature in both compartments was maintained throughout the experiments at the same levels, and set to mimic the seasonal outdoor temperatures of the area: 10°C at 0300h, increasing to 12°C at 0500h, 15°C at 0700h, and 20°C at 0900h, where it remained for 10 hours, before decreasing to 15°C at 1900h and 12°C at 2100h. Seedlings were watered as needed throughout the experiment, with the exception of the previously noted two days before and one day after treatment, with both compartments receiving the same frequency of watering. Seedlings were checked five days per week for visible aphids, which were removed either with tweezers or by spraying the branches of the seedling with high-pressure water. Any seedlings with aphids found on them were marked with flagging. On 26 June 2014, the aphid problem had spread so that it was unmanageable by manual removal, as most or all seedlings in both compartments were observed to have immature aphids and honeydew on their needles. As a result, seedlings were sprayed the following day with insecticidal soap (Safer^®^ Brand). No live aphids were found on any seedlings following this treatment.

### Sampling

All seedlings were sampled on 14 July 2014. Control and treatment seedlings were paired based on family and cone type, and then based on matching heights from the initial (pre-experiment measurements) as closely as possible. Paired control and treatment seedlings were sampled sequentially so that the treatment seedling was sampled, and then its corresponding control seedling was sampled immediately after. For each seedling, one branch was removed from the seedling at its base with clippers. The branch chosen was that which was located highest on the stem with at least some fully developed (grey) bark (previous year’s growth). The branch was packaged for chemical analysis after clipping it back from the tip to approximately 10 cm in length, removing all needles, and then placing it in a pre-labeled kraft paper envelope (Staples^®^ #1 coin envelopes, 5.7 cm x 8.9 cm), which was immediately placed onto dry ice. Immediately following sampling, the samples were packed in dry ice and shipped to the British Columbia Ministry of Environment, Knowledge Management Branch, North Road Laboratory, in Victoria, British Columbia for chemical analysis of terpenes, as well as chiral terpene analyses. All seedling heights were re-measured the day following the sampling procedure.

### Chemical analysis

Woody tissue samples were processed using gas chromatographic-flame ionization detection analyses, with standards matched to retention times to identify specific compounds. Two separate extraction procedures were used for the analysis. First, frozen samples (–80°C) were ground in liquid nitrogen, and an average of 0.39 g per sample was extracted for 48 h in 4 mL of hexane, with pentadecane (250 ppm) used as an internal standard. Next, samples were inverted and then allowed to settle for 24 h. One half a milliliter of solution was then transferred to a 2 mL autosampler vial for gas chromatographic analysis using a PerkinElmer Clarus580 (PerkinElmer, Waltham, Massachusetts) with built-in autosampler, with a ZB-WAXplus (Phenomenex, 30 m, 0.25 mm i.d., 0.25 μm film). The injection was split, at a rate of 16 mL/min, at approximately 12:1, and an injector temperature of 230°C. The carrier gas utilized was helium, at a pressure of 14.5 psi, a rate of 1.35 mL/min, at 60°C. For the temperature program, the oven temperature was initially held for one minute at 60°C, after which it was increased by 3.0°C/min to 85°C, then increased at a rate of 8.0°C/min to 170°C, and finally by 20.0°C/min to 250°C, where it was held for 12 min.

For the second extraction procedure, the same ground, frozen samples were extracted using 4 mL of methanol for 48 h, with 250 ppm pentadecane again used as an internal standard. Samples were then inverted and then allowed to settle for 24 h. Next, 0.5 mL of solution was transferred to a 2 mL autosampler vial for gas chromatographic analysis. A PerkinElmer Clarus580 (PerkinElmer, Waltham, Massachusetts) was used with built-in autosampler, with an ZB-5msi (Phenomenex, 30 m, 0.25 mm i.d., 0.25 μm film). The injection was split, at a rate of 23 mL/min, at approximately 20:1; and an injector temperature of 230°C. Helium was utilized as the carrier gas, with a pressure of 14.5 psi, a rate of 1.35 mL/min, at 60°C. The oven temperature was initially held at 60°C for 1 min, increased by 3.0°C/min to 85°C, then increased at a rate of 8.0°C/min to 170 °C, and finally increased by 20.0°C/min to 250°C and held for 12.0 min.

Concurrent with the hexane extraction, a separate portion of fresh woody tissue was weighed and oven-dried overnight at 70°C in order to remove residual moisture. An average of 0.35 g of tissue was used for this procedure, as with some samples there was a limited size of remaining sample. The dry mass obtained was used to determine a moisture correction, which was then applied to the results of both extraction procedures.

A total of approximately 33 terpenes were separated using this method. The methanol extraction procedure was found to extract more compound for each terpene than the hexane extractions, so methanol extraction values were used in the analysis. However, two compounds, limonene and β-phellandrene, were unable to be separated with the ZB-5 column which was used for the samples from the methanol extraction, so the data from the hexane extraction was used in the analysis for these two compounds. In addition, fenchone and terpinolene were not able to be differentiated by these methods, and so their concentrations are reported together, as “fenchone & terpinolene”.

### Chiral terpene analysis

A subsample of 45 of the original 150 samples was used for the analysis of chirality for five of the analyzed terpenes: β-pinene, α-pinene, camphene, limonene, and sabinene. The subsample was selected so that there were 15 samples of each cone type. Within each cone type, the samples selected were chosen randomly.

Samples were again analyzed by the British Columbia Ministry of Environment, Knowledge Management Branch, North Road Laboratory, using the previously obtained methanol extracts which, as already noted, showed a higher extraction efficiency when compared to the hexane extracts. Gas chromatographic-mass spectrometer detection analyses were used to process samples, and standards were matched to the retention time and mass spectrum of each compound. To carry out the procedure, approximately 0.5 ml of extract was placed in a 2 ml autosampler vial, and then analyzed using a Clarus 500 GC with a Claus 560 S mass spectrometer, with built in autosampler, which was fitted with a Cylcodex-B (Agilent, 30M, 0.25 mm id, 0.25 μm film) column. The injection was split (rate of 20 mL/min, approximately 10.3:1; injector temperature of 250°C), and the carrier gas utilized was helium (pressure 17 psi). For the temperature program, the oven temperature was initially held at 75°C for 15 min, then increased by 20.0°C/min to 230°C, where it was held for 15 min.

### Statistical analyses

All analyses were conducted in R version 3.0.2 [39]. All mixed effects ANOVA and ANCOVA models were fit using the “lme4” package [40], and were calculated using Type III (marginal) Sums of Squares. Contrasts between cone types, where overall effects were statistically significant (α = 0.05), were compared by least-squares mean differences using the “lmerTest” package [41].

### Effect of seed source and treatment type

To visually determine if the seedlings grown from different cone types could be separated based on their terpene profiles, a linear discriminant analysis was performed. Seedlings were grouped and analyzed separately for different sites and treatment types. Concentrations of all analyzed terpenes were used as the explanatory variables, while cone type was the pre-determined grouping variable. To satisfy the assumption of normality of the explanatory variables, all terpene concentrations were ln (x+1) transformed before analysis. The linear discriminant analysis was performed using the R “MASS” package [42].

Mixed effect analyses of variance (ANOVAs) were used to determine if there was an effect of the seed source (cone type) and the treatment on the concentration of each analyzed terpene, as well as the sum of the total terpenes, in the seedling woody tissue. Each analyzed terpene was considered as the dependent variable in an independent ANOVA. Fixed effects were “cone type” (seedlings grown from cones of dead trees, old cones on live trees or young cones on live trees), “treatment type” (MeJa treatment or control), and the potential two-way interaction between cone type and treatment type. The random effects were “site” (location of seed source) and “family” (maternal tree of seed source). The assumptions of homoscedasticity and normality of the residuals were assessed by plots of residuals. The dependent variables for all models were subsequently ln (x+1) transformed. In addition, Spearman’s rank correlation coefficients were calculated between the analyzed terpenes, with seedlings grouped by cone type of seed source and treatment type and correlations determined separately for each group, in order to determine the degree of relatedness of their production within the seedlings.

### Chiral terpene analysis

To determine if the seed source and/or MeJa treatment had an effect on the proportion of (+) and (–) enantiomers of β-pinene, α-pinene, camphene, limonene, and sabinene within the seedlings, mixed effects ANOVAs were used. The factors used were the same as those for the above terpene ANOVAs: fixed factors of cone type, treatment type, and the interaction between cone type and treatment type, as well as random factors for site and family. The response variable was the proportion of the (+) enantiomer for each sample, and was ln (x) transformed prior to analysis.

### Growth rates of seedlings

The growth of the seedlings over the course of the experiments was analyzed by using mixed effects Analysis of Covariance (ANCOVA). The dependent variable was the total height growth (cm) of the seedlings during the experiment. Fixed factors were those that were considered for the terpene ANOVAs: cone type, treatment type, and the interaction between cone type and treatment type. In addition, a covariate for initial height of the seedling was included. The random effects were site and family. No transformation was required to satisfy the assumptions of the test, based on residual plots.

To directly assess if there appeared to be a tradeoff between terpene production and growth rates, a Pearson’s product-moment correlation test was used to determine if there was a significant relationship between the concentration of total terpenes produced and the total height growth of each seedling.

## Results

### Chemical composition of seedlings

Ten terpenes (β-phellandrene, β-pinene, δ-3-carene, α-pinene, camphene, myrcene, limonene, sabinene, α-phellandrene, and fenchone & terpinolene) were predominant in the lodgepole pine seedling woody tissue, with mean concentrations of >150 ppm, each accounting for at least 1% of the total terpenes. A further thirteen terpenes (ocimene, bornyl acetate, geranyl acetate, linalool, α-terpinene, p-cymene, β-thujone, β-caryophyllene, γ-terpinene, α-thujone, α-thujene, citronellal, and borneol) were found in moderate concentrations (mean >10 ppm), each representing >0.1% of the total. A final seven terpenes and (citronellol, citronellyl acetate, terpineol, geraniol, camphor, α-humulene, and pulegone) were found in trace amounts only, each representing ≤ 0.1% of the total. For further details on the terpene concentrations, see Table 2.

**Table 2:** Descriptive statistics of concentrations of analyzed terpenes in lodgepole pine woody tissue.

Terpene	Mean concentration (ppm)	Standard Error (ppm)	Mean %
β-Phellandrene	4694	179	37.3
β-Pinene	3399	229	27.0
δ-3-Carene	1072	63	8.52
α-Pinene	1010	50	8.02
Camphene	526	14	4.18
Myrcene	329	14	2.62
Limonene	301	24	2.39
Sabinene	261	27	2.07
α-Phellandrene	201	19	1.60
Fenchone & Terpinolene	157	15	1.25
Ocimene	113	16	0.90
Bornyl acetate	91	5	0.72
Geranyl acetate	78	10	0.62
Linalool	52	2	0.42
β-Thujone	42	11	0.33
α-Terpinene	40	5	0.32
p-Cymene	40	3	0.32
γ-Terpinene	35	1	0.28
β-Caryophyllene	35	7	0.28
α-Thujone	32	9	0.26
α-Thujene	21	5	0.16
Citronellal	15	4	0.12
Borneol	14	1	0.11
Citronellol	9	2	0.07
Citronellyl acetate	8	1	0.06
Terpineol	4	1	0.04
Geraniol	2	0.7	0.01
Camphor	2	0.6	0.01
α-Humulene	1	0.7	0.01
Pulegone	1	0.3	0.004
2-Carene	0	0	0.00
1,8-Cineol	0	0	0.00
Citronellene	0	0	0.00
Total	12584	719	100

N = 150

### Effect of seed source on overall terpene profiles on seedlings

**Fig 2:**
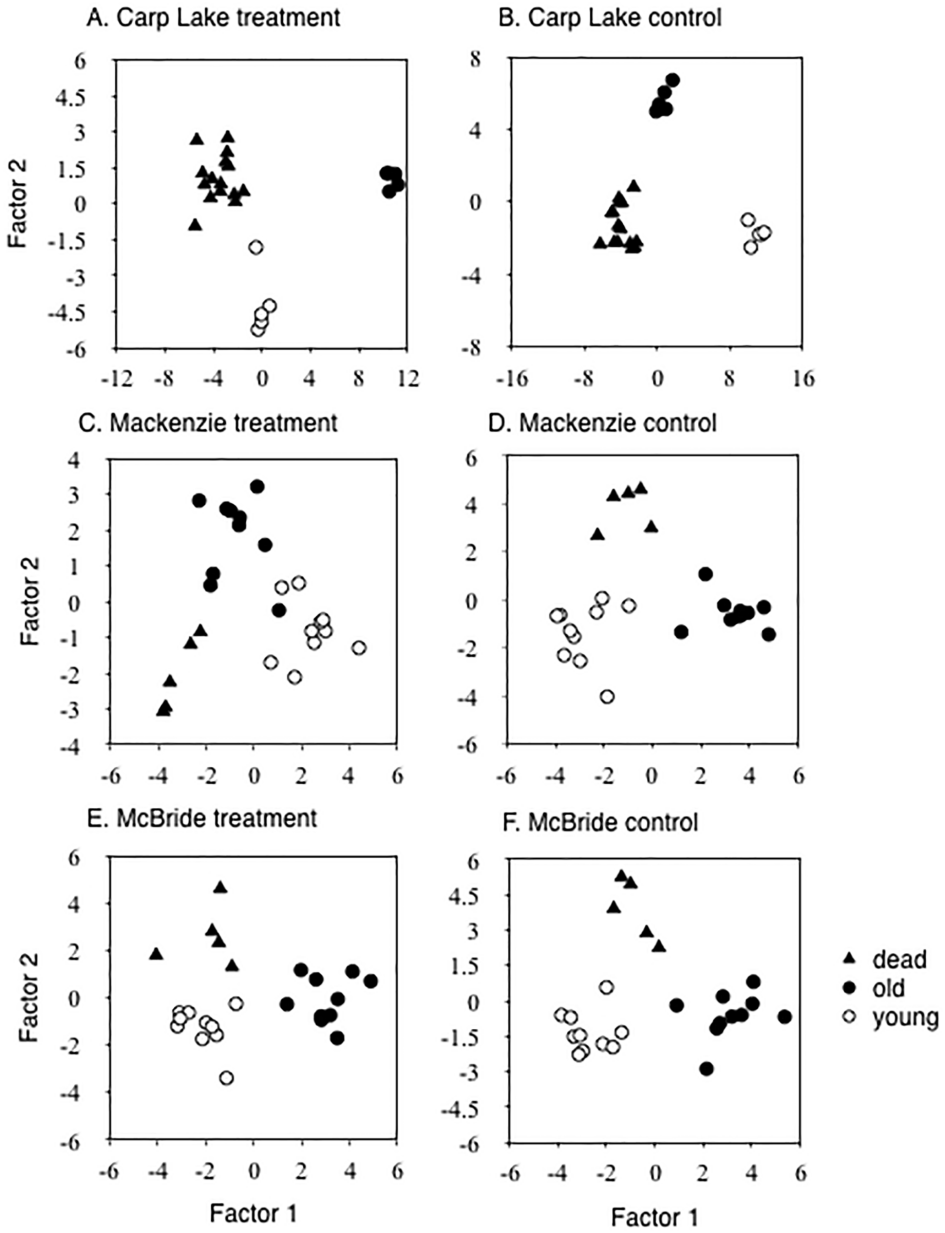
Canonical scores plots from linear discriminant analysis, showing the separation of cone types by total terpene concentrations. Concentrations of all cone types were ln (x+1) transformed before analysis.

The linear discriminant analysis showed distinct separation based on the terpene profiles of the seedlings between those grown from different cone types (Fig 2). Seedlings grown from the Carp Lake seed source separated clearly by cone type (Fig 2A and 2B). Those grown from either the Mackenzie or McBride seed source separated by cone type without or with minimal overlap based on their terpene profiles, but were less clearly differentiated than those from Carp Lake (Fig 2C–2F).

### Effect of seed source and treatment type

There were significant differences in the production of ten different terpenes between seedlings grown from seeds of different maternal cone types, shown in Table 3. These were myrcene, sabinene, fenchone & terpinolene, ocimene, α-terpinene, p-cymene, α-thujene, citronellol, gerianol, and camphor. In addition, limonene, citronellal, and borneol showed possible but non-significant trends in concentration based on cone type of seed source. Finally, the total concentration of analyzed terpenes was not significantly different for the seedlings grown from the different cone types.

**Table 3:** Results of the mixed effects ANOVAs for the effect of MeJa treatment and conetype of maternal seed source on terpene production in woody tissue of lodgepole pine seedlings.

Terpene	Conetype			Treatment			Conetype*Treatment		
	df	*F*	*P*	df	*F*	*P*	df	*F*	*P*
β-Phellandrene	2,144	0.30	0.74	1,144	2.33	0.13	2,144	1.15	0.32
β-Pinene	2,19.6	1.15	0.34	1,136.4	1.00	0.32	2,136.4	0.51	0.60
δ-3-Carene	2,29.4	0.24	0.79	1,137	0.19	0.66	2,137	0.97	0.38
α-Pinene	2,20.6	1.26	0.30	1,135.2	0.44	0.51	2,135.2	0.03	0.97
Camphene	2,28.4	1.60	0.22	1,134.8	0.00	0.99	2,134.8	0.38	0.69
Myrcene	**2,144**	**4.68**	**0.01**	1,144	0.05	0.83	2,144	0.16	0.85
Limonene	2,134	2.85	0.06	1,142	0.72	0.40	1,142	1.18	0.31
Sabinene	**2,143.5**	**8.00**	**<0.001**	1,142	0.02	0.89	2,142	0.49	0.61
α-Phellandrene	2,30.6	0.93	0.41	1,135.4	0.09	0.76	2,135.4	0.63	0.54
Fenchone & Terpinolene	**2,143.7**	**5.21**	**0.007**	1,142	0.00	0.97	2,142	2.06	0.13
Ocimene	**2,19.2**	**5.79**	**0.01**	**1,136.2**	**4.25**	**0.04**	2,136.2	0.51	0.60
Bornyl acetate	2,32.5	0.64	0.53	1,136.3	0.18	0.67	2,136.3	0.12	0.89
Geranyl acetate	2,17.3	1.77	0.20	1,136.3	1.28	0.26	2,136.3	0.97	0.38
Linalool	2,126.8	0.99	0.37	**1,142**	**4.05**	**0.046**	2,142	0.02	0.98
β-Thujone	2,143.6	1.72	0.18	1,142	0.39	0.53	2,142	0.11	0.89
α-Terpinene	**2,32**	**4.50**	**0.02**	1,136.1	0.17	0.68	2,136.1	0.11	0.90
p-Cymene	**2,116.3**	**4.94**	**0.009**	1,142.2	0.41	0.52	2,142.2	0.25	0.78
γ-Terpinene	2, 40.3	0.03	0.97	1,138.1	0.93	0.34	2,138.1	0.08	0.93
β-Caryophyllene	2,24.6	0.62	0.55	1,136.1	0.51	0.48	2,136.1	1.15	0.32
α-Thujone	2,120.9	0.01	0.99	1,141,8	0.05	0.82	2,141,8	0.02	0.98
α-Thujene	**2,141.5**	**9.80**	**<0.001**	1,142	1.13	0.29	2,142	0.14	0.87
Citronellal	2,18.1	3.01	0.07	1,135.9	0.44	0.51	2,135.9	2.06	0.13
Borneol	2,19.4	3.46	0.052	1,130.5	0.01	0.92	2,130.5	0.10	0.91
Citronellol	**2,30.7**	**3.67**	**0.04**	1,135.9	0.28	0.59	2,135.9	1.66	0.19
Citronellyl acetate	2,36.8	1.76	0.19	1,137.2	2.08	0.15	2,137.2	0.05	0.95
Terpineol	2,22.9	1.08	0.36	1,137.3	1.77	0.19	2,137.3	0.14	0.87
Geraniol	**2,144**	**3.80**	**0.02**	1,144	0.00	0.99	2,144	0.30	0.74
Camphor	**2,34.2**	**6.44**	**0.004**	1,137.2	0.38	0.54	2,137.2	0.22	0.80
α-Humulene	2,14.2	0.33	0.73	1,135.3	1.04	0.31	2,135.3	0.05	0.95
Pulegone	2,128.4	1.88	0.16	1,142.1	0.00	0.97	2,142.1	0.00	1.00
Total	2,20.4	1.41	0.27	1,135.9	2.52	0.11	2,135.9	0.48	0.62

Random effects were site and family, df = degrees of freedom (numerator, denominator). Statistically significant effects are highlighted in bold text.

For most of the terpenes that were present in moderate to high concentrations in the seedlings, contrasts of the different cone types showed a similar general trend, summarized in Table 4. The lowest terpene concentrations were those in the woody tissue of the seedlings grown from cones on dead trees, moderate amounts from those grown from old cones on live trees, and the highest amounts in the seedlings grown from young cones on live trees. However, not all pairs of contrasts were significant for all compounds. For four of the terpenes (myrcene, sabinene, fenchone & terpinolene, and ocimene), there were significant differences between the seedlings grown from dead trees compared to live trees, but no significant differences between the old and young cones on the live trees. One additional terpene (p-cymene) showed differences between the cones from dead trees and young cones from live trees, while the old cones from live trees were intermediate in concentrations and not significantly different from either. Finally, a single terpene (α-terpinene) was found in higher concentrations in the seedlings grown from the young cones compared to either those grown from old cones or dead trees, which were not significantly different from one another.

**Table 4:** Effect of cone type of seed source on concentrations (ppm, mean±1 SE) of terpenes in lodgepole pine woody tissue.

Terpene	Conetype		
	Dead	Old	Young
Myrcene	278±14*a*	335±15*b*	376±37*b*
Sabinene	172±13*a*	272±32*b*	338±71*b*
Fenchone+Terpinolene	118±13*a*	162±19*b*	192±39*b*
Ocimene	30±7*a*	136±19*b*	174±43*b*
a-Terpinene	30±2*a*	33±2*a*	58±15*b*
p-Cymene	33±2*a*	35±2*ab*	53±10*b*
a-Thujene	16±2*a*	22±2*b*	24±16*a*
Citronellal	8±4*a*	29±10*a*	8±3*a*
Borneol	9±1*a*	15±2*ab*	18±2*b*
Citronellol	5±3*a*	15±4*b*	6±2*a*
Geraniol	0.3±0.3*a*	5±2*b*	0.5±0.5*a*
Camphor	0.4±0.3*a*	4±2*b*	0*a*

Different letters indicate significant differences (α = 0.05) among cone types (mixed effects ANOVA followed by least-squares mean differences with the “lmerTest” package in R [41]).

The terpenes found in trace amounts in the seedlings, generally showed a different trend (Table 4). The seedlings grown from old cones of live trees generally showed the highest concentrations, while those grown from cones from dead trees and those grown from young cones on live trees were generally similar and lower. For three of the terpenes (citronellol, geraniol, and camphor), the concentrations in seedlings grown from old cones were significantly higher than either those from the dead trees or the new cones. In addition, α-thujene followed this same trend, although it was present in slightly greater quantities than the rest of the trace terpenes.

Comparisons of MeJa treated seedlings with control seedlings showed significant differences for only two terpenes, shown in Tables 3 and 5. There were significantly higher levels of ocimene, and lower levels of linalool for MeJa treated seedlings compared to control seedlings. There were no significant differences between the concentrations of total measured terpenes in treatment seedlings and compared to control seedlings, and there were no significant interaction effects between cone type and treatment type for any of the measured terpenes (Table 3).

**Table 5:** Terpene concentrations (ppm, mean±1 SE) of lodgepole pine seedlings, comparing seedlings treated with a MeJa solution and control seedlings.

Terpene	Treatment	Control
Ocimene	137±29*a*	89±15*b*
Linalool	40±3*a*	56±3*b*
Total Terpenes	13094±525*a*	12075±499*a*

The differences in ocimene and linalool concentrations were statistically significant (α = 0.05), while the differences in total terpene concentrations were not (mixed effects ANOVA followed by least-squares mean differences with the “lmerTest” package in R [41]).

The significance tests of the random effects (Table 6) suggested a significant effect of site of seed source for fenchone & terpinolene, and a possible but non-significant trend between sites of seed source for β-thujone. In addition, there were significant overall effects of family of seed source for β-pinene, δ-3-carene, α-pinene, ocimene, geranyl acetate, β-caryophyllene, citronellal, terpineol, α-humulene, and total terpene concentrations.

**Table 6:** Significance tests of the random effects (site and family) from the mixed effects ANOVAs for the effect of MeJa treatment and conetype of maternal seed source on terpene production in woody tissue of lodgepole pine seedlings.

Terpene	Site		Family	
	χ ^2^_(1)_	*P*	χ^2^_(1)_	*P*
β-Phellandrene	0.00	1.00	0.00	1.00
β-Pinene	1.11	0.29	**5.98**	**0.01**
δ-3-Carene	0.00	1.00	**6.01**	**0.01**
α-Pinene	0.01	0.94	**4.89**	**0.03**
Camphene	0.00	1.00	1.50	0.20
Myrcene	0.00	1.00	0.00	1.00
Limonene	0.73	0.40	0.00	1.00
Sabinene	1.61	0.20	0.00	1.00
α-Phellandrene	1.12	0.30	0.00	1.00
Fenchone & Terpinolene	**5.23**	**0.02**	0.00	1.00
Ocimene	0.00	1.00	**14.00**	**<0.001**
Bornyl acetate	0.00	1.00	2.33	0.10
Geranyl acetate	0.66	0.42	**7.78**	**0.005**
Linalool	0.29	0.60	0.00	1.00
β-Thujone	3.56	0.06	0.00	1.00
α-Terpinene	0.19	0.70	0.53	0.50
p-Cymene	0.10	0.80	0.00	1.00
γ-Terpinene	0.00	1.00	0.50	0.50
β-Caryophyllene	0.00	1.00	**4.43**	**0.04**
α-Thujone	0.20	0.70	0.00	1.00
α-Thujene	1.58	0.20	0.00	1.00
Citronellal	0.03	0.86	**6.57**	**0.01**
Borneol	0.00	1.00	0.16	0.70
Citronellol	0.00	1.00	0.21	0.60
Citronellyl acetate	0.00	1.00	1.97	0.20
Terpineol	1.14	0.29	**4.25**	**0.04**
Geraniol	0.00	1.00	0.00	1.00
Camphor	0.01	0.90	1.50	0.20
α-Humulene	0.00	1.00	**20.70**	**<0.001**
Pulegone	0.41	0.50	0.00	1.00
Total	0.00	1.00	**7.17**	**0.007**

Statistically significant effects are highlighted in bold text.

The Spearman’s rank correlations between the concentrations of the measured terpenes in the seedlings showed a number of significant, moderate-to-strong positive correlations, with correlation coefficients ≥ 0.60. For the full correlation tables, see S1A–S1F Table. Myrcene, camphene, and α-phellandrene were all positively correlated with one another. For these three terpenes, maximum pairwise correlation coefficients were 0.91 for myrcene with camphene (old cones, control), 0.92 for myrcene with α-phellandrene (dead cones, control) and 0.95 for camphene with α-phellandrene (dead cones, control). Other notable strong positive correlations were fenchone & terpinolene with sabinene (*r* ranging from 0.55 to 0.82), and α-pinene with β-pinene (*r* ranging from 0.60 to 0.81), both of which were significantly correlated for seedlings grown from all cone types and treatment types. Although the majority of the correlations between terpene concentrations were positive, there were also a few significant negative correlations observed. The highest of these negative correlations was between linalool and β-caryophyllene among the seedlings grown from young cones in the control group (*r* = -0.56). Additional notable negative correlations found amongst the seedlings grown from old cones were β-pinene with borneol (*r* = -0.53) and linalool with geraniol (*r* = -0.55) in the treatment group, as well as β-caryophyllene with α-thujene (*r* = -0.52) in the control group. All other negative correlations were weaker, with |*r*|<0.50.

A comparison of the terpene correlations between the different groups of seedlings based on cone type of seed source and treatment type suggested that there may be some general trends. For both the seedlings grown from young cones from live trees and from dead trees, there was a significantly higher number of significant correlations for the control seedlings than for the treatment seedlings (young cones: 38 significant correlations for treatment seedlings vs. 85 correlations for control seedlings, ***χ***^***2***^_***(1)***_ = 17.96, ***P***<0.001, dead trees: 55 significant correlations for treatment seedlings vs. 92 correlations for control seedlings, ***χ***^***2***^_***(1)***_ = 9.31, ***P*** = 0.002). There was no difference in the number of significant correlations between terpenes for the seedlings grown from the old cones (57 for treatment seedlings and 53 for control seedlings, ***χ***^***2***^_***(1)***_ = 0.15, ***P*** = 0.70). However, for seedlings grown from all three cone types, the maximum correlation coefficient was higher for the control seedlings than for the treatment seedlings (young = 0.89 for control, 0.80 for treatment, old = 0.91 for control, 0.87 for treatment, and dead = 0.95 for control, 0.93 for treatment). Overall, the terpenes in the seedlings grown from the young cones in the treatment group appeared to be the least correlated, with a total of only 38 significant correlations, with a maximum correlation coefficient of .80, while the seedlings grown from the old cones in the control group appeared to be the most correlated, with a total of 92 significant correlations and a maximum correlation coefficient of 0.95.

### Chiral terpene analysis

Descriptive statistics of the analyzed chiral terpenes are shown in Table 7. For β-pinene, the (+)-enantiomer was present in greater concentrations than that of the (–)-enantiomer, and represented 72% of the total β-pinene concentration. For α-pinene, camphene, and sabinene, the (–)-enantiomer was predominant, and represented 83%, 62%, and 73% of the enantiomeric blend, respectively. Ratios of the (+)- and (–)-enantiomers for limonene were similar.

**Table 7:** Descriptive statistics and comparisons by paired *t*-tests of the proportion of concentrations of (+)- and (–)-enantiomers of analyzed terpenes in lodgepole pine woody tissue. N = 45.

Chiral terpene	(+) Enantiomer			(-) Enantiomer		Mean %	*t*_44)_	*P*
	Mean (ppm)	Std. Err. (ppm)	Mean %	Mean (ppm)	Std. Err. (ppm)			
β-Pinene	1280	158	72	508	50	28	4.32	**<0.001**
α-Pinene	288	36	17	1420	73	83	-14.89	**<0.001**
Camphene	27	3	38	43	4	62	-3.00	**0.004**
Limonene	286	46	59	199	45	41	1.27	0.21
Sabinene	108	19	27	298	24	73	-5.66	**<0.001**

Statistically significant effects are highlighted in bold text.

The mixed effects ANOVAs, describing the effect of the cone type of maternal seed source and treatment type on the proportion of the (+)-enantiomer, did not show a significant difference of the effects of either of the fixed effects predictor variables or their interaction (Table 8). For the analysis of the random effects, there was a significant effect of site of seed source on the proportion of the (+)-enantiomer of β-pinene (Table 9).

**Table 8:** Results of the mixed effects ANOVAs for the effect of MeJa treatment and cone type of maternal seed source on the proportion of (+)-enantiomer produced for five chiral terpenes in woody tissue of lodgepole pine seedlings.

Chiral terpene	Conetype			Treatment			Conetype*Treatment		
	df	*F*	*P*	df	*F*	*P*	df	*F*	*P*
β-Pinene	2,37.6	1.35	0.27	1,37.3	0.58	0.45	2,37.1	2.34	0.11
α-Pinene	2,16	0.25	0.78	1,32.5	0.34	0.57	2,32.3	0.12	0.89
Camphene	2,15.7	0.53	0.60	1,34.7	0.00	0.97	2,34.3	0.18	0.84
Limonene	2,38.1	1.00	0.38	1,37.6	2.92	0.096	2,37.2	0.71	0.50
Sabinene	2,38.2	0.49	0.62	1,47.6	0.18	0.67	2,37.2	0.12	0.89

Random effects were site and family, df = degrees of freedom (numerator, denominator), N = 45.

**Table 9:** Significance tests of the random effects (site and family) from the mixed-model ANOVAs for the effect of MeJa treatment and cone type of maternal seed source on the proportion of (+)-enantiomer produced for five chiral terpenes in woody tissue of lodgepole pine seedlings.

Chiral terpene	Site		Family	
	χ ^2^_(1)_	*P*	χ^2^_(1)_	*P*
β-Pinene	3.97	0.05	0.00	1
α-Pinene	0.00	1	0.34	0.6
Camphene	0.25	0.6	0.03	0.9
Limonene	0.14	0.7	0.00	1
Sabinene	0.28	0.6	0.00	1

N = 45.

### Growth rates of seedlings

The mixed effects ANCOVA for the effect of the categorical predictors on the total height growth of the seedlings showed no significant effects for any of the fixed effects: cone type (*F*_(2,21.9_ = 0.45, *P* = 0.64), treatment (*F*_(1,134.1)_ = 0.0002, *P* = 0.99), or the interaction of cone type and treatment (*F*_(2,133.7)_ = 0.56, *P* = 0.57), or either of the random effects: site (χ ^2^_(1)_ = 0.30, *P* = 0.6), or family (χ ^2^_(1)_ = 0.99, *P* = 0.3). In addition, there was not a significant correlation between height growth and total terpene production in the seedlings (*r* = -0.092, *P* = 0.26).

## Discussion

### Effect of seed source and treatment type

Seedlings grown from parental seed sources that had survived the MPB attack by varying amounts (as represented by cone types) showed differential production of terpenes (as represented by their overall terpene profiles), as well as a differential production of a number of individual terpenes. In all cases, where there were differential concentrations of individual terpenes between seedlings grown from different cone types, the seedlings grown from the cones from live trees (either old and/or young cones) produced higher terpene concentrations than those grown from the cones of dead trees. For the major, predominant terpenes, the seedlings grown from young cones on live trees generally had the highest concentrations, followed by those from the old cones on live trees, and finally the lowest levels in the seedlings grown from dead trees. In contrast, a significant induced defensive reaction from the MeJa treatment was not detected for the majority of terpenes. This may be because MeJa does not induce a defensive reaction in the same manner, or as extensively, as actual MPB infestation [20]. In addition, previous work has found that resistant trees undergoing induced defense had higher levels of both limonene and terpinolene than those that were less resistant [32, 43]. In this study, we observed increased levels of fenchone & terpinolene (combined concentrations) in seedlings grown from resistant cone classes, as well as a possible but non-significant difference in limonene concentrations. Further, we observed extensive aphid feeding on the majority of seedlings. This may suggest that the majority of the seedlings in this study were under at least some stress and exhibiting some level of induced defenses, which may have affected both the quality and quantity of terpenes produced.

Where significant differences existed between seedlings grown from different seed sources, the terpenes present in trace amounts showed a somewhat different pattern than that of the predominant terpenes. For these trace compounds, the seedlings grown from the old cones on live trees produced the highest concentrations, which were higher even than those grown from the young cones on the same trees. Some of these trace compounds are similar in chemical composition or structure, or are linked to the synthesis of other, more prevalent terpenes which follow the more expected pattern described above. For example, geraniol and linalool, as well as α-thujene and sabinene, are both structural isomers of one another. In addition, the terpenes showing this trend, except α-thujene, were oxygenated compounds (alcohols or ketones), and therefore might be produced as the result of modifications of other more prevalent terpenes. For example, the oxidation of the alcohol borneol produces the ketone camphor [44], while Kännaste [45] suggested that the synthesis of the alcohol gernaniol may be linked to both limonene and myrcene, which were predominant monoterpenes in our seedlings. Further, many of the trace terpenes were negatively correlated with more prevalent terpenes, especially in the seedlings grown from the old cones. For example, there were significant negative correlations between camphor and sabinene, geraniol and p-cymene, as well as α-thujene and β-caryophyllene in the control seedlings; and, borneol and β-pinene, camphor and fenchone & terpinolene, as well as geraniol and linalool in the MeJa-treated seedlings. If the production of these compounds elicited some additional defense against the MPB or other insects or pathogens, we would expect they would give those seedlings with a tendency to produce them in large quantities an advantage over those that did not (i.e., over those grown from the seeds of dead cones). However, if their production came at an overall energetic cost, such as reducing the production of the more predominant terpenes, which may confer even more defensive qualities, those trees would be at a disadvantage compared to those which produced less of them (i.e. those grown from the seeds of young cones).

Lodgepole pine defensive reactions appear to be highly generalized, increasing concentrations of all produced terpenes simultaneously [32, 33]. However, previous work has suggested that the production of certain individual terpenes as a result of induced defenses, including δ-3-carene and β-pinene, is highly heritable [31]. Therefore, qualitative differences in the quantities of individual terpenes produced, such as those seen in our study, are likely due to heritable tendencies to produce differential amounts of specific terpenes, within the context of this generalized defensive response. The particular terpenes which showed differences in expression between seedlings from resistant vs. non-resistant parents did not precisely follow the pattern expected for MPB-specific resistance. For example, limonene, δ-3-carene, and α-pinene have all been found to be toxic to MPB eggs. Raffa and Berryman [33] noted that prolonged exposure to 1% concentrations of these monoterpenes caused 60%, 40% and 37% egg mortality, respectively, compared to 10% for control eggs. In addition, Erbilgin *et al*. [46] found significantly higher concentrations of limonene and 3-carene in lodegpole pine trees that were attacked but survived the MPB compared to unattacked trees. However, in our study, none of these monoterpenes differed significantly by cone type. Conversely, Erbilgin *et al*. [46] found higher levels of both myrcene and terpinolene in unattacked compared to attacked but surviving lodgepole trees. In our study, we found that both myrcene and terpinolene levels were higher in seedlings from surviving trees, which is therefore not consistent with the expected results from a MPB- defense standpoint.

The observed differences from the expected MPB-defensive phytochemistry of the seedlings can be explained by several factors. First, due to ontogenetic changes over time, ratios of conifer terpenes change as the trees age, and thus the ratios of terpenes found within seedlings would not be fully representative of the terpene profiles of these trees at maturity when they would be subjected to MPB pressure. These changes in defensive compounds as trees mature may be due to a number of factors, including developmental limitations in young plants, changing priorities in resource allocation as plants age, multiple functions of certain plant defensive chemicals, and changes in herbivore pressure over time [47]. This final possibility is particularly important to this study, as the primary herbivores of young lodgepole pine trees (e.g. terminal and root collar weevils, mites, aphids, cutworm larvae) differ substantially from those of older trees (bark beetles including MPB and other *Dendroctonus*, *Ips*, and *Hylurgops* spp.) [48]. In addition, differences in defensive chemicals produced between the different tissues of the trees should be expected, as previous work has found differences between the ratios of monoterpenes in foliar vs. cortical tissue for several conifer species including lodgepole pine [49, 4]. This is highly relevant, as we measured the terpene concentrations in branch tips, while MPB attacks the bole of the tree. Finally, the method of induction of defensive compounds (MeJa) may not precisely mimic those defenses induced by MPB attack, as Burke *et al*. [20] found that the quantity and composition of terpenes produced by the same tree varied between MeJa treatment compared to mechanical wounding combined with fungal inoculant. Regardless of these limitations, the appearance of qualitative changes in terpene expression within a single lodgepole pine generation suggest that the severe beetle pressure such as that seen in the interior of British Columbia may create a strong natural selective sieve for these tendencies.

### Chiral terpene analysis

For camphene, sabinene, and α-pinene, the seedlings consisted of predominantly the (–)-enantiomer. These results support those of Pureswaran *et al*. [4], who found similar trends of camphene and sabinene, and those of Clark *et al*. [50] who found similar trends for α-pinene in lodgepole pine woody tissue. In addition, the (–)-enantiomer of α-pinene is the molecular form that is converted to the biologically active enantiomer of the pheromone (–) *trans*-verbenol by MPB females [9]. There was no significant difference between the (+) and (–) forms of limonene in the seedlings analyzed in this study. Conversely, previous work on mature trees has suggested that (–)-limonene tends to be more prevalent than the (+) form [4, 50]. Further, the seedlings used in this study appeared to express predominantly the (+)-enantiomer of β-pinene, rather than the (–)-form. This contrasts with the results of Pureswaran *et al*. [4], who found that the (–)-enantiomer was more abundant in both the bole woody tissue (bark, phloem and sapwood) and foliage of mature trees, and the results of Clark *et al*. [50] who found only the (–)-enantiomer and none of the (+)-enantiomer in the phloem tissue of mature trees. The differences observed between this study and previous work in these two terpenes may be due to using seedlings instead of mature trees, as enantiomeric ratios of certain terpenes can change as some conifer seedlings age [45]. Additionally, differences may be observed since we used branches instead of woody tissue from the bole of the tree, as previous studies have shown that chiral monoterpene compositions vary between different tissues within the same tree [4, 49. Alternatively, the result may represent regional differences in enantiomeric compositions—for instance for β-pinene, due to the differences observed due to the random factor of site, or an adaptive difference due to resistance traits. However, there was no observed difference in proportions of enantiomers between different cone types or treatment types for any of the chiral terpenes measured, indicating that there is either no adaptive effect, a single generation is insufficient, or the scope of the study is inadequate to detect an effect of chirality.

### Growth rates of seedlings

The short-term growth rates of the seedlings suggested that in this study, there was no apparent effect of cone type or treatment type on growth, or apparent direct trade-off between terpene production and growth. This parallels the results of Raffa and Berryman [32], who found no differences in previous five-year growth rates for mature lodgepole pine trees that were classified as resistant or susceptible to MPB. Conversely, Ferrenberg *et al*. [51] found that lodgepole pine trees that were MPB resistant tended to have higher radial and basal area increment growth rates than those trees that were killed by the MPB, though the radial growth trend was not significant. Additionally, several *Pinus* spp. have shown a tradeoff between induced defenses elicited by MeJa and growth rates, with increased defensive metabolite production reducing growth [52–54]. These results may indicate that the overall elicitation of induced defenses come at a cost, but the specific genetic-based defenses that increase resistance to the MPB are less costly, and thus might confer a direct fitness benefit.

### Limitations and conclusions

There are several important limitations of this study. First, the study had a fairly small sample size of maternal trees. In addition, the study utilized seedlings which were only three years of age, and only the branch defensive chemistry was tested, which may not be representative of the terpene profiles of the bole of a mature tree, which would be the MPB’s target tissue. Further, the presence of the aphids was unfortunate, as there was no real control for the induction of defenses. Finally, since existing, previously pollinated cones were used, we were unable to directly control the pollination source of any of the cone types and thus in some cases, the pollinators may be different than presumed.

Despite these limitations, this study does support a number of general conclusions. The study suggests that phenotypic traits that determine resistance to the MPB do have a heritable effect on the physiology of terpenes produced by offspring seedlings. Further, given that there were no, or at most minimal, observed tradeoffs in growth and defensive terpene production, these results provide evidence that selection for phenotypes that may be more resistant to the MPB could have net positive effects. Therefore, selection for resistance traits is an advisable option for replanting of pine forests in the wake of the most destructive natural force in recent years. Further study is needed to determine which terpenes are the most important for MPB resistance, and to what extent the ability to synthesize these terpenes in biologically relevant amounts and timing can be passed down to future generations.

## Supporting information

S1 Table Spearman’s rank correlation coefficients for all analyzed terpenes, separated by cone type and by treatment type, showing only those which were significantly different from zero at α = .05 (|r|>0.40). βPh = β-phellandrene, βPi = β-Pinene, δ3C = δ-3-Carene, αPi = α-Pinene, Cam = Camphene, Myr = Myrcene, Lim = Limonene, Sab = Sabinene, αPh = α-Phellandrene, FT = Fenchone & Terpinolene, Oci = Ocimene, BA = Bornyl acetate, GA = Geranyl acetate, Lin = Linalol, βTh = β-Thujone, αTer = α-Terpinene, pCy = p-Cymene, γTe = γ-Terpinene, βCa = β = Citronellal, αTho = α-Thujone, αThe = α-Thujene, Cta = Citronellal, Bor = Borneol, Cto = Citronellol, CA = Citronellyl acetate, Ter = Terpineol, Ger = Geraniol, Cmr = Camphor, αHu = α-Humulene, Pul = Pulegone.
(DOCX)
