# Supplementary material for: Single-generation effects on terpenoid defenses in lodgepole pine populations following mountain pine beetle infestation
Source: PLoS One. 2018 May 14;13(5):e0196063. doi: 10.1371/journal.pone.0196063 (PMC5951554; doi:10.1371/journal.pone.0196063)
Supplement: S1 Table — βPh = β-phellandrene, βPi = β-Pinene, δ3C = δ-3-Carene, αPi = α-Pinene, Cam = Camphene, Myr = Myrcene, Lim = Limonene, Sab = Sabinene, αPh = α-Phellandrene, FT = Fenchone & Terpinolene, Oci = Ocimene, BA = Bornyl acetate, GA = Geranyl acetate, Lin = Linalol, βTh = β-Thujone, αTer = α-Terpinene, pCy = p-Cymene, γTe = γ-Terpinene, βCa = β = Citronellal, αTho = α-Thujone, αThe = α-Thujene, Cta = Citronellal, Bor = Borneol, Cto = Citronellol, CA = Citronellyl acetate, Ter = Terpineol, Ger = Geraniol, Cmr = Camphor, αHu = α-Humulene, Pul = Pulegone. (DOCX) [file pone.0196063.s001.docx]

## Supplemental Material

A. Young cone seed source, MeJa treatment

|  | βPh | βPi | δ3C | αPi | Cam | Myr | Lim | Sab | αPh | FT | GA | Lin | βTh | αTe | γTe | αThe |
| --- | --- | --- | --- | --- | --- | --- | --- | --- | --- | --- | --- | --- | --- | --- | --- | --- |
| αPi |  | .69 | .45 | - |  |  |  |  |  |  |  |  |  |  |  |  |
| Cam | .51 |  |  | .63 | - |  |  |  |  |  |  |  |  |  |  |  |
| Myr |  |  | .47 | .57 | .73 | - |  |  |  |  |  |  |  |  |  |  |
| Sab |  |  | .40 |  |  | .66 |  | - |  |  |  |  |  |  |  |  |
| αPh | .41 |  |  | .43 | .80 | .72 |  |  | - |  |  |  |  |  |  |  |
| FT |  |  | .51 |  |  | .52 |  | .80 |  | - |  |  |  |  |  |  |
| Oci |  |  |  |  |  | .41 |  | .44 |  |  |  |  |  |  |  |  |
| GA | .47 |  |  |  |  |  |  |  |  |  | - |  |  |  |  |  |
| pCy |  |  |  | .42 | .70 | .52 |  |  | .46 |  |  | .42 |  | .51 |  |  |
| γTe |  |  | .49 |  |  | .40 |  | .53 |  | .59 |  |  |  |  | - |  |
| βCa |  |  |  |  |  |  |  |  |  |  |  |  |  |  |  |  |
| αTho |  |  |  |  |  |  |  |  |  |  |  |  | .65 |  | -.41 |  |
| αThe |  |  |  |  |  |  |  |  |  | .49 | .52 |  |  |  |  | - |
| Bor |  |  |  |  |  |  |  |  | .41 |  |  |  |  |  |  |  |
| CA |  |  |  |  |  |  |  |  |  | -.47 |  |  |  |  |  | -.41 |
| Ter |  |  |  |  |  |  |  |  |  | .49 |  |  |  |  |  | .55 |

B. Young cone seed source, control

|  | βPh | βPi | δ3C | αPi | Cam | Myr | Lim | Sab | αPh | FT | BA | GA | Lin | βTh | αTe | pCy | γTe | βCa | Cta | Bor | Cito | CA |
| --- | --- | --- | --- | --- | --- | --- | --- | --- | --- | --- | --- | --- | --- | --- | --- | --- | --- | --- | --- | --- | --- | --- |
| βPi | -.40 | - |  |  |  |  |  |  |  |  |  |  |  |  |  |  |  |  |  |  |  |  |
| αPi |  | .64 |  | - |  |  |  |  |  |  |  |  |  |  |  |  |  |  |  |  |  |  |
| Cam | .72 | -.40 |  |  | - |  |  |  |  |  |  |  |  |  |  |  |  |  |  |  |  |  |
| Myr | .67 |  | .59 | .52 | .60 | - |  |  |  |  |  |  |  |  |  |  |  |  |  |  |  |  |
| Lim | .54 |  | .40 | .55 |  | .63 | - |  |  |  |  |  |  |  |  |  |  |  |  |  |  |  |
| Sab | .45 |  |  | .40 | .43 | .74 |  | - |  |  |  |  |  |  |  |  |  |  |  |  |  |  |
| αPh | .70 | -.41 | .42 |  | .65 | .82 |  | .74 | - |  |  |  |  |  |  |  |  |  |  |  |  |  |
| FT |  |  | .46 | .51 |  | .56 | .42 | .78 | .41 | - |  |  |  |  |  |  |  |  |  |  |  |  |
| Oci |  |  |  |  |  | .42 |  |  |  |  |  |  |  |  |  |  |  |  |  |  |  |  |
| BA |  |  | .48 |  |  | .40 |  |  | .54 |  | - |  |  |  |  |  |  |  |  |  |  |  |
| GA | .45 | -.47 |  |  |  | .44 |  | .46 | .55 |  |  | - |  |  |  |  |  |  |  |  |  |  |
| αTe |  |  |  |  |  | .42 |  | .49 | .66 |  |  | .41 | .40 |  | - |  |  |  |  |  |  |  |
| pCy | .52 | -.48 |  |  | .80 | .46 |  |  | .62 |  |  |  |  |  | .67 | - |  |  |  |  |  |  |
| γTe | .43 |  | .47 |  |  | .59 |  | .58 | .71 | .55 |  | .42 |  |  | .82 | .58 | - |  |  |  |  |  |
| βCa |  |  |  |  |  |  |  |  |  |  |  |  | -.56 |  |  |  | -.40 | - |  |  |  |  |
| αTho |  |  |  |  |  |  |  |  |  |  |  |  |  | .89 |  |  |  |  |  |  |  |  |
| Cta |  |  |  |  |  |  |  |  |  |  | .42 |  |  |  |  |  |  |  | - |  |  |  |
| Bor | .40 |  |  |  |  | .42 | .41 |  | .42 |  |  | .53 | .60 |  | .43 | .49 | .58 | -.46 |  | - |  |  |
| Cito |  |  |  |  |  |  |  |  |  |  |  | .42 |  |  |  |  |  |  | .83 |  | - |  |
| CA |  |  |  |  |  |  |  |  |  |  | .40 | .40 |  |  |  |  |  |  | .56 |  | .71 | - |
| Ter | .50 |  |  |  | .40 | .51 | .58 |  |  |  |  |  |  |  |  |  |  |  |  | .49 |  |  |
| Ger |  |  |  |  |  |  |  |  |  |  |  |  |  |  |  |  |  |  | .55 |  | .66 | .44 |

C. Old cone seed source, MeJa treatment

|  | βPh | βPi | δ3C | αPi | Cam | Myr | Lim | Sab | αPh | FT | BA | GA | Lin | βTh | αTe | βCa | Cta | Cito |
| --- | --- | --- | --- | --- | --- | --- | --- | --- | --- | --- | --- | --- | --- | --- | --- | --- | --- | --- |
| αPi |  | .81 |  | - |  |  |  |  |  |  |  |  |  |  |  |  |  |  |
| Cam | .70 |  |  | .66 | - |  |  |  |  |  |  |  |  |  |  |  |  |  |
| Myr | .62 |  |  | .52 | .87 | - |  |  |  |  |  |  |  |  |  |  |  |  |
| Lim | .47 |  |  |  | .61 | .53 | - |  |  |  |  |  |  |  |  |  |  |  |
| Sab |  |  |  |  |  | .56 |  | - |  |  |  |  |  |  |  |  |  |  |
| αPh | .66 |  |  |  | .81 | .85 | .48 | .50 | - |  |  |  |  |  |  |  |  |  |
| FT |  |  | .40 |  |  |  |  | .55 |  | - |  |  |  |  |  |  |  |  |
| Oci |  |  | -.41 |  |  |  |  |  |  |  |  |  |  |  |  |  |  |  |
| BA |  |  |  |  |  | .49 |  | .61 | .51 |  | - |  |  |  |  |  |  |  |
| GA |  |  |  |  |  | .47 |  |  |  |  |  | - |  |  |  |  |  |  |
| αTe |  |  |  |  |  | .40 |  |  | .64 |  |  |  | .54 |  | - |  |  |  |
| pCy |  |  |  |  | .41 | .53 |  | .52 | .60 |  | .68 |  |  |  | .57 |  |  |  |
| γTe |  |  | .43 |  |  | .46 |  | .66 |  | .72 |  |  |  |  | .50 |  |  |  |
| βCa |  |  |  |  |  |  |  |  |  |  |  | .41 |  |  |  | - |  |  |
| αTho |  |  |  |  |  |  |  |  |  |  |  |  |  | .71 |  |  |  |  |
| αThe | .45 |  |  |  |  |  |  |  | .41 |  |  |  |  |  |  |  |  |  |
| Cta |  |  | .44 |  |  |  |  |  |  |  |  | .40 |  |  |  | .41 | - |  |
| Bor |  | -.53 |  |  |  |  |  |  |  |  |  |  |  |  |  | .40 |  |  |
| Cito |  |  |  |  |  |  |  |  |  |  |  | .49 |  |  |  | .52 | .80 | - |
| CA |  |  |  |  |  |  |  |  |  |  |  | .68 |  |  |  |  | .70 | .65 |
| Ter |  |  |  |  | .40 | .41 |  |  |  |  |  |  |  |  |  |  |  |  |
| Ger |  |  |  |  |  |  |  |  |  |  |  |  | -.55 |  |  |  |  |  |
| Cmr |  |  |  |  | .47 | .47 |  |  |  | -.42 |  |  |  |  |  |  |  |  |

D. Old cone seed source, control

|  | βPh | βPi | δ3C | αPi | Cam | Myr | Lim | Sab | αPh | FT | Oci | GA | βTh | αTe | pCy | βCa | αTho | Cta | Cito | CA | Ter | Ger | Cmr |
| --- | --- | --- | --- | --- | --- | --- | --- | --- | --- | --- | --- | --- | --- | --- | --- | --- | --- | --- | --- | --- | --- | --- | --- |
| αPi |  | .81 |  | - |  |  |  |  |  |  |  |  |  |  |  |  |  |  |  |  |  |  |  |
| Cam | .41 |  |  | .64 | - |  |  |  |  |  |  |  |  |  |  |  |  |  |  |  |  |  |  |
| Myr |  |  |  | .52 | .91 | - |  |  |  |  |  |  |  |  |  |  |  |  |  |  |  |  |  |
| Lim |  |  |  |  | .64 | .63 |  |  |  |  |  |  |  |  |  |  |  |  |  |  |  |  |  |
| Sab |  |  |  |  |  |  |  | - |  |  |  |  |  |  |  |  |  |  |  |  |  |  |  |
| αPh | .62 |  |  |  | .78 | .79 | .54 |  | - |  |  |  |  |  |  |  |  |  |  |  |  |  |  |
| FT |  |  |  |  |  |  |  | .82 |  | - |  |  |  |  |  |  |  |  |  |  |  |  |  |
| Oci |  |  |  |  |  |  |  |  |  |  | - |  |  |  |  |  |  |  |  |  |  |  |  |
| BA |  |  |  |  | .48 | .47 |  |  | .55 |  |  |  |  |  |  |  |  |  |  |  |  |  |  |
| GA | .40 |  |  |  |  |  |  |  |  |  |  | - |  |  |  |  |  |  |  |  |  |  |  |
| pCy |  |  |  |  | .45 | .40 |  |  | .41 |  |  |  |  | .65 | - |  |  |  |  |  |  |  |  |
| γTe |  |  | .63 |  |  |  |  | .43 |  | .44 |  |  |  | .44 |  |  |  |  |  |  |  |  |  |
| βCa |  | .48 |  |  |  |  |  |  |  |  | .51 |  |  |  |  | - |  |  |  |  |  |  |  |
| αTho |  |  |  |  |  |  |  |  |  |  |  |  | .74 |  |  |  | - |  |  |  |  |  |  |
| αThe | .59 |  |  |  |  |  |  | .55 | .55 | .56 |  |  |  |  |  | -.52 |  |  |  |  |  |  |  |
| Cta |  | .47 |  | .50 |  |  |  |  |  |  |  |  |  |  |  |  |  | - |  |  |  |  |  |
| Bor |  |  |  | .42 |  |  |  |  |  |  |  |  | .48 |  |  |  |  |  |  |  |  |  |  |
| Cito |  |  |  |  |  |  |  |  |  |  |  | .66 |  |  |  |  |  | .53 | - |  |  |  |  |
| CA |  |  |  |  |  |  |  | -.44 |  |  |  | .60 |  |  |  |  |  |  | .49 | - |  |  |  |
| Ter |  |  |  |  |  |  |  |  |  |  |  |  |  |  |  |  |  |  |  |  | - |  |  |
| Ger |  | .40 |  |  |  |  |  |  |  |  |  |  |  | -.48 | -.41 |  |  |  |  |  |  | - |  |
| Cmr |  |  |  |  |  |  |  | -.47 |  |  |  | .43 |  |  |  |  |  |  | .64 | .45 |  |  | - |
| αHu |  |  |  |  |  |  |  |  |  |  |  |  |  |  |  |  |  |  |  |  |  | .50 |  |
| Pul |  |  |  |  |  |  |  |  |  |  |  |  |  |  |  |  | .45 |  |  | .41 | .72 |  | .44 |

E. Dead tree seed source, MeJa treatment

|  | βPh | βPi | δ3C | αPi | Cam | Myr | Sab | αPh | FT | BA | Lin | βTh | αTe | pCy | βCa | Cta | CA |
| --- | --- | --- | --- | --- | --- | --- | --- | --- | --- | --- | --- | --- | --- | --- | --- | --- | --- |
| αPi |  | .75 |  | - |  |  |  |  |  |  |  |  |  |  |  |  |  |
| Cam | .45 | .51 |  | .84 | - |  |  |  |  |  |  |  |  |  |  |  |  |
| Myr | .56 |  | .48 |  | .70 | - |  |  |  |  |  |  |  |  |  |  |  |
| Lim |  |  |  |  |  |  |  |  |  |  |  |  |  |  |  |  |  |
| Sab | .55 |  | .58 | .40 | .60 | .93 | - |  |  |  |  |  |  |  |  |  |  |
| αPh | .65 |  |  | .42 | .65 | .86 | .88 | - |  |  |  |  |  |  |  |  |  |
| FT |  |  | .92 |  |  | .54 | .59 |  | - |  |  |  |  |  |  |  |  |
| BA |  |  |  |  | .42 |  |  | .43 |  | - |  |  |  |  |  |  |  |
| Lin |  |  |  |  |  |  |  |  |  | .54 | - |  |  |  |  |  |  |
| βTh |  |  |  |  |  |  |  |  |  | .44 |  | - |  |  |  |  |  |
| αTe |  |  |  |  |  | .42 | .40 | .40 | .48 |  |  |  | - |  |  |  |  |
| pCy |  |  |  |  |  |  |  |  |  |  | .51 | .44 |  | - |  |  |  |
| γTe |  |  | .68 |  |  | .45 | .52 |  | .70 |  |  |  | .49 |  |  |  |  |
| βCa | .42 |  |  | .43 | .51 | .65 | .54 | .54 |  |  |  |  |  |  | - |  |  |
| αTho |  |  |  |  |  |  |  |  |  |  |  | .74 |  | .45 |  |  |  |
| αThe |  |  |  |  |  |  |  | .42 |  |  |  |  |  |  |  |  |  |
| Bor | .40 |  |  |  |  | .49 | .61 | .61 |  |  |  |  |  |  |  | .43 |  |
| CA |  |  |  |  |  | .53 | .49 |  |  |  |  |  |  |  | .47 |  | - |
| Ter |  |  |  |  |  |  |  |  |  |  |  |  |  |  |  |  | .64 |
| αHu |  |  |  |  |  |  |  |  |  | .43 |  |  |  |  | .45 |  |  |

F. Dead tree seed source, control

|  | βPh | βPi | δ3C | αPi | Cam | Myr | Lim | Sab | αPh | FT | Oci | BA | GA | Lin | βTh | αTe | pCy | γTe | βCa | αTho | αThe | Cta | Cito | Ter |
| --- | --- | --- | --- | --- | --- | --- | --- | --- | --- | --- | --- | --- | --- | --- | --- | --- | --- | --- | --- | --- | --- | --- | --- | --- |
| αPi |  | .60 |  | - |  |  |  |  |  |  |  |  |  |  |  |  |  |  |  |  |  |  |  |  |
| Cam | .66 |  |  | .56 | - |  |  |  |  |  |  |  |  |  |  |  |  |  |  |  |  |  |  |  |
| Myr | .68 |  |  | .50 | .87 | - |  |  |  |  |  |  |  |  |  |  |  |  |  |  |  |  |  |  |
| Lim | .57 |  |  | .64 | .45 | .42 | - |  |  |  |  |  |  |  |  |  |  |  |  |  |  |  |  |  |
| Sab | .52 |  | .56 |  | .55 | .66 |  | - |  |  |  |  |  |  |  |  |  |  |  |  |  |  |  |  |
| αPh | .71 |  |  | .50 | .95 | .92 | .44 | .66 | - |  |  |  |  |  |  |  |  |  |  |  |  |  |  |  |
| FT |  |  | .70 |  |  |  |  | .72 |  | - |  |  |  |  |  |  |  |  |  |  |  |  |  |  |
| BA |  |  |  |  | .63 | .67 |  | .54 | .66 |  |  | - |  |  |  |  |  |  |  |  |  |  |  |  |
| GA |  |  |  |  | .43 | .44 |  |  |  |  | .70 |  | - |  |  |  |  |  |  |  |  |  |  |  |
| Lin |  |  |  |  | .51 |  |  |  | .43 |  |  |  |  | - |  |  |  |  |  |  |  |  |  |  |
| αTe |  |  |  |  | .82 | .76 |  | .50 | .79 |  |  | .60 |  | .64 |  | - |  |  |  |  |  |  |  |  |
| pCy |  |  | .52 |  | .50 | .50 |  | .66 |  | .43 |  | .40 |  | .53 |  | .56 | - |  |  |  |  |  |  |  |
| γTe |  |  | .51 |  | .52 | .60 |  | .73 | .63 | .70 |  |  |  |  |  | .58 | .46 | - |  |  |  |  |  |  |
| βCa |  |  |  |  | .54 |  |  |  | .57 |  |  |  |  |  |  | .41 |  |  | - |  |  |  |  |  |
| αTho |  |  |  |  |  |  |  |  |  |  |  | -.43 |  |  | .72 |  |  |  |  | - |  |  |  |  |
| αThe | .56 |  |  |  | .46 | .50 | .53 | .46 | .55 | .41 |  | .41 |  |  |  |  |  | .65 |  |  | - |  |  |  |
| Cta |  |  |  |  |  |  |  |  |  |  | .46 |  | .56 |  |  |  |  |  |  |  |  | - |  |  |
| Bor | .62 |  |  |  | .55 | .51 | .42 | .54 | .51 |  |  |  |  |  |  |  |  |  |  |  |  |  |  |  |
| Cito |  |  |  |  |  |  |  |  |  |  |  |  | .47 |  |  |  |  |  |  |  |  | .87 | - |  |
| CA |  |  |  |  |  |  |  |  |  |  |  |  | .50 |  |  |  |  |  |  | .51 |  |  |  |  |
| Ter | .44 |  |  |  | .51 | .45 |  |  | .56 |  |  |  |  |  | .50 |  |  |  | .45 |  |  |  |  | - |
| Cmr |  |  |  |  |  |  |  |  |  |  |  |  |  |  |  |  |  |  |  |  |  | .58 | .70 |  |
| αHu |  |  |  |  | .41 |  |  |  | .47 |  |  |  |  |  |  |  |  |  | .49 |  | .43 |  |  | .76 |
